# When mechanical power remains high despite conventional lung-protective settings: a physiology-driven bedside framework

**DOI:** 10.1016/j.aicoj.2026.100095

**Published:** 2026-05-28

**Authors:** Tomasz Urbankowski, Marek Darowski

**Affiliations:** Department of Modeling and Supporting of Internal Organs Functions, Nalecz Institute of Biocybernetics and Biomedical Engineering, Polish Academy of Sciences, ul. Ks. Trojdena 4, 02-109 Warsaw, Poland

**Keywords:** Mechanical power, Mechanical ventilation, Ventilator-induced lung injury, End-expiratory lung volume, Prone positioning

## Abstract

Mechanical power integrates ventilatory pressure, tidal volume, flow, and respiratory rate into a single estimate of the energy transferred to the respiratory system per unit time. Higher mechanical power has been associated with ventilator-induced lung injury and worse outcomes. A common bedside dilemma arises when mechanical power remains high despite conventional lung-protective settings, because further reduction of ventilator intensity may compromise gas exchange.

This narrative, physiology-driven review addresses how to respond when persistently elevated mechanical power may reflect physiological constraint rather than easily modifiable ventilator settings. We outline a bedside approach in which clinicians first verify conventional lung-protective settings and then look for the main physiological factor that prevents further ventilator reduction. Reduced end-expiratory lung volume should prompt individualized positive end-expiratory pressure (PEEP) titration aimed at restoring and stabilizing lung volume while avoiding overdistension and derecruitment. In mechanically heterogeneous lungs, prone positioning and patient-specific pressure optimization may improve the physiological conditions associated with injurious ventilation, even when the global value of mechanical power cannot be safely lowered. During assisted ventilation, excessive respiratory drive and inspiratory effort should be assessed and treated when present. When high mechanical power remains unavoidable despite optimized settings and targeted physiological interventions, extracorporeal support may be required to enable ultra-protective ventilation.

## Highlights

• Persistently high mechanical power may indicate physiological constraint.
• Reduced EELV can sustain injurious ventilation despite optimized settings.
• Prone positioning and individualized PEEP may reduce regional stress.
• In assisted ventilation, patient effort may amplify hidden lung stress.
• Extracorporeal support may enable safer ultra-protective ventilation.

## Background

Mechanical power (MP) integrates ventilatory pressure, tidal volume, flow, and respiratory rate into a single estimate of the energy delivered to the respiratory system per unit time [1]. By capturing both static and dynamic components of ventilatory load, it provides an integrative measure of the mechanical burden imposed on the lung [1]. Its determinants include tidal volume, driving pressure, respiratory rate, inspiratory flow, and, in some formulations, positive end-expiratory pressure (PEEP); however, their relative contributions are not equivalent [1].

From a practical perspective, MP is not yet uniformly displayed by ventilators, and its calculation depends on ventilator mode and on the variables available at the bedside [1,2]. In some formulations, its estimation additionally requires conditions such as passive inflation and plateau pressure measurement [1,3]. Accordingly, MP is best understood not as a universally available standalone bedside target, but as a decision-support parameter that helps integrate the physiological significance of its major components [1,3]. Even when formal MP calculation is not readily available, clinically accessible variables—particularly driving pressure, tidal volume, and respiratory rate—can still support the same physiology-based reasoning [1,2].

The elastic dynamic component, largely reflected by driving pressure and tidal volume in relation to compliance, is generally considered the most biologically relevant, whereas the contribution of resistive power related to flow and airway resistance remains more controversial, because a substantial fraction of this energy may be dissipated in the airways and endotracheal tube rather than in the lung parenchyma itself [4,5]. In keeping with this, Costa et al. showed that a simplified model combining driving pressure and respiratory rate performed similarly to formal MP estimation in association with mortality in acute respiratory distress syndrome (ARDS) [2].

Mechanical power should be interpreted as an unscaled global surrogate of ventilatory energy transfer rather than as a direct measure of regional tissue injury [4,5]. It becomes physiologically and clinically meaningful only when interpreted in the context of functional lung size and regional heterogeneity, because the same global MP may correspond to very different patterns of regional stress, strain, and energy dissipation across lungs of different size and mechanical structure [[4], [5], [6]].

The elastic component is likely to remain the primary focus of bedside interpretation, because it is more closely linked to lung stress, strain, and compliance-related load, whereas the significance of resistive power is less directly linked to tissue injury and may be more context-dependent [4,5]. However, resistive components should not be considered negligible in all circumstances. In heterogeneous lungs and under high-flow conditions, especially when accompanied by patient–ventilator dyssynchrony or strong inspiratory effort, excessive inspiratory flow may contribute to regional shear stress, pendelluft amplification, flow-dependent inhomogeneity, and greater dynamic transpulmonary pressure swings, thereby potentially increasing injurious local stress even when the global MP value changes little [5,[7], [8], [9]]. This distinction also guides the clinical framework developed in the subsequent sections: in controlled ventilation, bedside interpretation focuses primarily on elastic constraints related to reduced functional lung volume and regional heterogeneity, whereas in assisted ventilation greater attention must be paid to situations in which patient effort, dyssynchrony, or excessive inspiratory flow amplify dynamic transpulmonary stress.

**Fig. 1:**
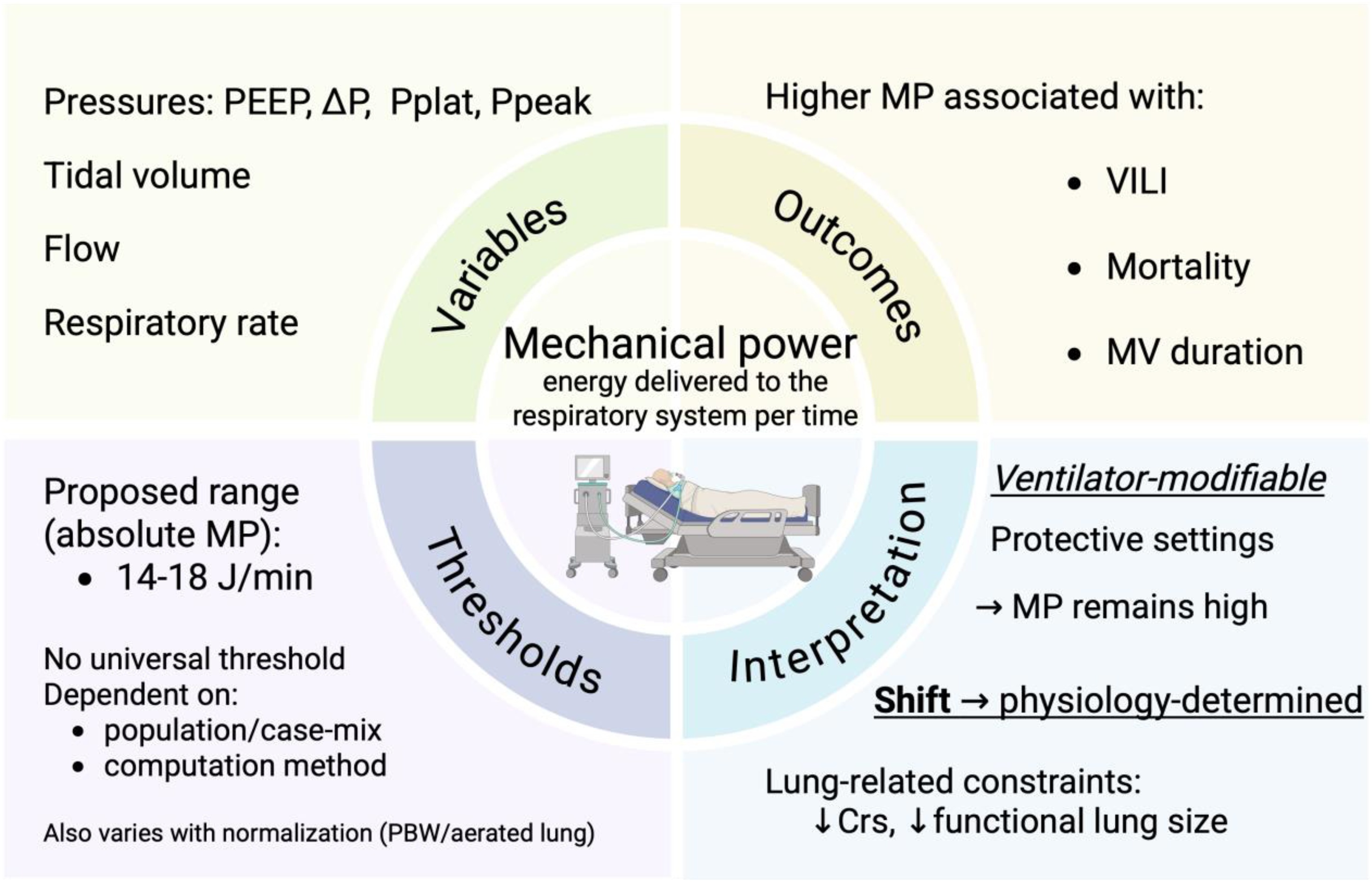
Mechanical power (MP) summarizes pressures, tidal volume, flow, and respiratory rate as energy delivered to the respiratory system per unit time. Higher MP is linked to VILI, mortality, and longer MV duration. Proposed absolute risk ranges often cluster around 14–18 J/min, but vary by population, computation method, and normalization. The figure also illustrates persistently high MP despite conventional lung-protective settings, consistent with lung-related constraints (↓Crs, ↓functional lung size). ΔP = driving pressure; Crs = compliance of the respiratory system; MP = mechanical power; MV = mechanical ventilation; PBW = predicted body weight; PEEP = positive end-expiratory pressure; Ppeak = peak inspiratory pressure; Pplat = plateau pressure; VILI = ventilator-induced lung injury.

Higher MP has been associated with ventilator-induced lung injury and worse clinical outcomes, and proposed risk thresholds most often cluster around approximately 14–18 J/min [10]. Importantly, these values represent population-level associations rather than patient-specific injury thresholds, and their applicability at the individual bedside level is limited by substantial inter-individual variability in lung size, mechanics, and susceptibility to injury. Fig. 1 summarizes the principal determinants of MP, its associations with adverse outcomes, and the clinical scenario in which MP remains high despite otherwise conventional protective settings.

A key clinical challenge arises when MP remains elevated despite conventional lung-protective settings. In such cases, further reduction of ventilatory intensity—particularly reductions in tidal volume, respiratory rate, or PEEP—may lower MP numerically but at the cost of inadequate oxygenation or ventilation. Persistently high MP may therefore indicate that the injured lung—functionally small, consistent with the classical “baby lung” concept—cannot safely tolerate further energy delivery under conventional settings [4,6].

Here, we use this concept to organize a practical bedside approach for controlled and assisted ventilation, focusing on the dominant modifiable mechanisms: reduced or unstable EELV, regional heterogeneity, excessive inspiratory effort, and persistent gas-exchange demands.

*Literature approach:* This article is a narrative, physiology-driven review and does not represent a systematic review or formal evidence synthesis. The literature was identified through focused searches in PubMed/MEDLINE and Embase, supplemented by backward and forward citation tracking of seminal articles, key physiological papers, and recent reviews relevant to the clinical problem addressed in this manuscript.

Search strategies combined controlled vocabulary terms (MeSH in PubMed and Emtree in Embase) and free-text keywords related to mechanical power, ventilator-induced lung injury, acute respiratory distress syndrome, end-expiratory lung volume, derecruitment, lung heterogeneity, prone positioning, respiratory drive, inspiratory effort, patient self-inflicted lung injury, and extracorporeal support, using Boolean operators (AND, OR) to refine topic-specific searches. Representative search combinations included terms such as “mechanical power” AND (“acute respiratory distress syndrome” OR ARDS) AND (“ventilator-induced lung injury” OR VILI), as well as topic-specific combinations for PEEP, prone positioning, respiratory drive, and extracorporeal support.

We preferentially considered articles that were mechanistically informative or clinically relevant to the bedside problem of persistently elevated mechanical power despite conventional lung-protective settings, including physiological studies, experimental work, observational clinical studies, major trials, and selected guideline or review papers when they clarified the clinical context. No formal protocol, prespecified systematic search strategy, study flow diagram, or risk-of-bias assessment was used. Conference abstracts without sufficient methodological detail, articles not directly relevant to invasive mechanical ventilation in acute respiratory failure or ARDS, and papers with limited relevance to the physiological framework of this review were not prioritized.

Therefore, the resulting framework should be interpreted as an integrative and interpretative bedside synthesis informed by selected literature, rather than as a systematic or exhaustive review of all available evidence. The selected literature was used to support key physiological principles and to inform a practical bedside decision framework, while recognizing that the evidence underlying the discussed mechanisms and interventions ranges from physiological and mechanistic rationale to clinical outcome data. Where outcome data are limited, the proposed strategies should be regarded as physiology-based, hypothesis-guided approaches requiring individualized bedside reassessment and confirmation in future clinical studies.

## When further reduction of ventilatory intensity becomes unsafe during controlled ventilation

In controlled ventilation, there is a physiological point at which further reduction of ventilatory intensity becomes counterproductive. At that stage, persistently high MP no longer reflects simply excessive ventilator settings, but limited tolerance of the injured lung to ongoing energy delivery because of reduced functional lung volume, derecruitment, regional heterogeneity, or their combination [4,11,12].

Further lowering tidal volume, respiratory rate, or PEEP may then produce only a small arithmetical reduction in MP while worsening gas exchange, promoting derecruitment, and increasing unstable opening and closing of lung units [5,13,14]. In mechanically heterogeneous lungs, it may also favor pendelluft, that is, movement of gas from one lung region to another within the same breath, thereby increasing local energy dissipation despite a lower global MP [5,9].

**Fig. 2:**
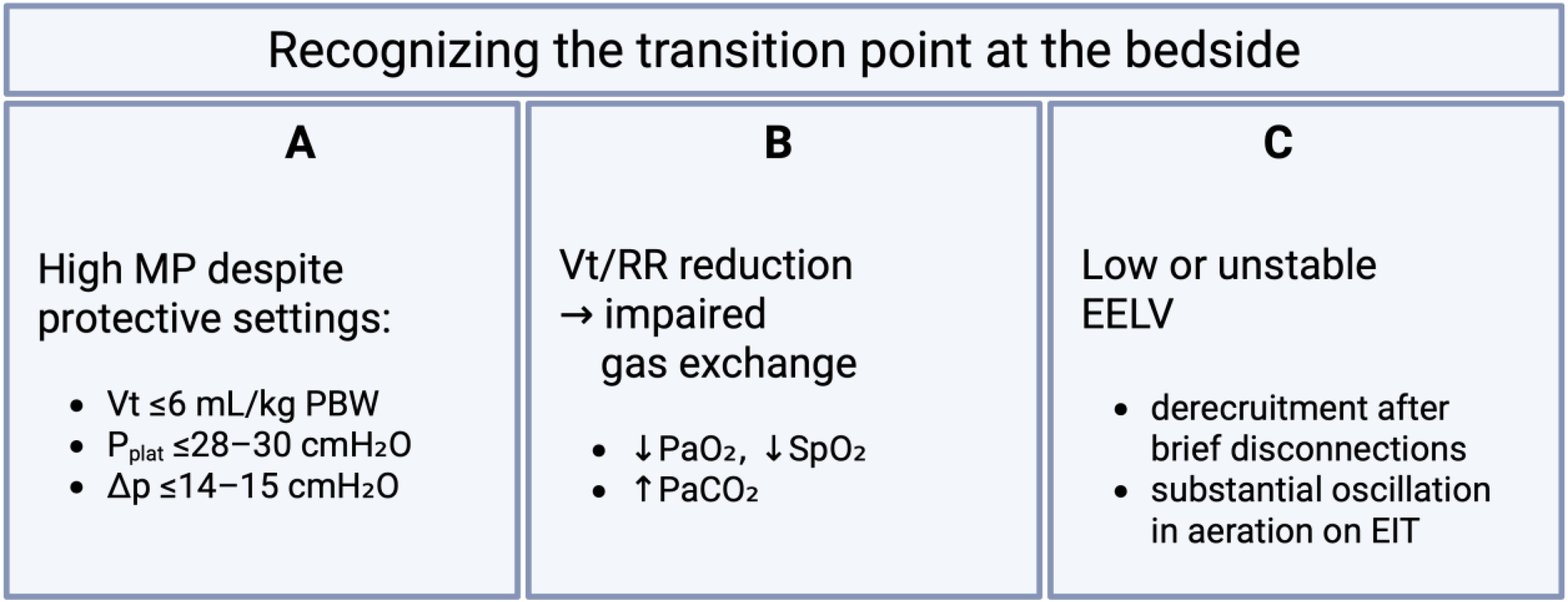
This figure summarizes pragmatic bedside indicators suggesting a transition from predominantly ventilator-modifiable mechanical power (MP) to a physiology-determined phase in which further reduction of ventilatory intensity is poorly tolerated. (A) MP remains high despite conventional lung-protective settings (Vt ≤6 mL/kg PBW, Pplat ≤28–30 cmH_2_O, Δp ≤14–15 cmH_2_O). (B) Further reductions in Vt and/or RR impair gas exchange, reflected by declining oxygenation (↓PaO₂ and/or ↓SpO_2_) and/or rising CO₂ (↑PaCO_2_). (C) Low or unstable EELV is suggested by derecruitment after brief disconnections and/or substantial oscillation in regional aeration on electrical impedance tomography (EIT). When these features coexist, persistently high MP is more likely driven by lung pathology and limited recruitable/functional lung volume rather than by modifiable ventilator settings. Δp = driving pressure; EELV = end-expiratory lung volume; EIT = electrical impedance tomography; MP = mechanical power; PaCO_2_ = arterial partial pressure of carbon dioxide; PaO_2_ = arterial partial pressure of oxygen; PBW = predicted body weight; P_plat_ = plateau pressure; RR = respiratory rate; SpO_2_ = peripheral oxygen saturation; Vt = tidal volume

From a practical bedside perspective, the next step should no longer be continued numerical minimization of MP at any cost, but identification of the dominant physiological mechanism that makes further ventilator reduction poorly tolerated [3,15]. Pragmatic indicators of this transition include: (1) persistently elevated MP despite already optimized conventional lung-protective settings (tidal volume ≤6 mL/kg predicted body weight, plateau pressure ≤28–30 cmH₂O, driving pressure ≤14–15 cmH₂O) [16,17]; (2) clinically relevant deterioration in oxygenation and/or development of hypercapnia or respiratory acidosis when tidal volume is further reduced [[18], [19], [20]] and (3) evidence that EELV is low or unstable, such as worsening mechanics or oxygenation after PEEP reduction, circuit disconnection, suctioning, or other derecruiting events, or marked oscillation in aeration on electrical impedance tomography [[21], [22], [23]]. These bedside features are summarized in Fig. 2.

Once this pattern is recognized, the next step is to identify whether poor tolerance of further ventilatory reduction is driven predominantly by loss or instability of end-expiratory lung volume, marked regional heterogeneity with uneven stress distribution, or both, because each mechanism implies a different therapeutic response [24,25]. When reduced or unstable EELV appears dominant, individualized PEEP titration and strict avoidance of derecruiting events should be prioritized [22,26]. When regional heterogeneity is the main concern, strategies that improve ventilation homogeneity and reduce regional stress concentration, particularly prone positioning, should be favored [[27], [28], [29]]. When both coexist, treatment should be individualized according to the dominant physiological mechanism, with reassessment after each therapeutic adjustment [26,29].

## Restoring end-expiratory lung volume to mitigate injurious ventilatory conditions

**Fig. 3:**
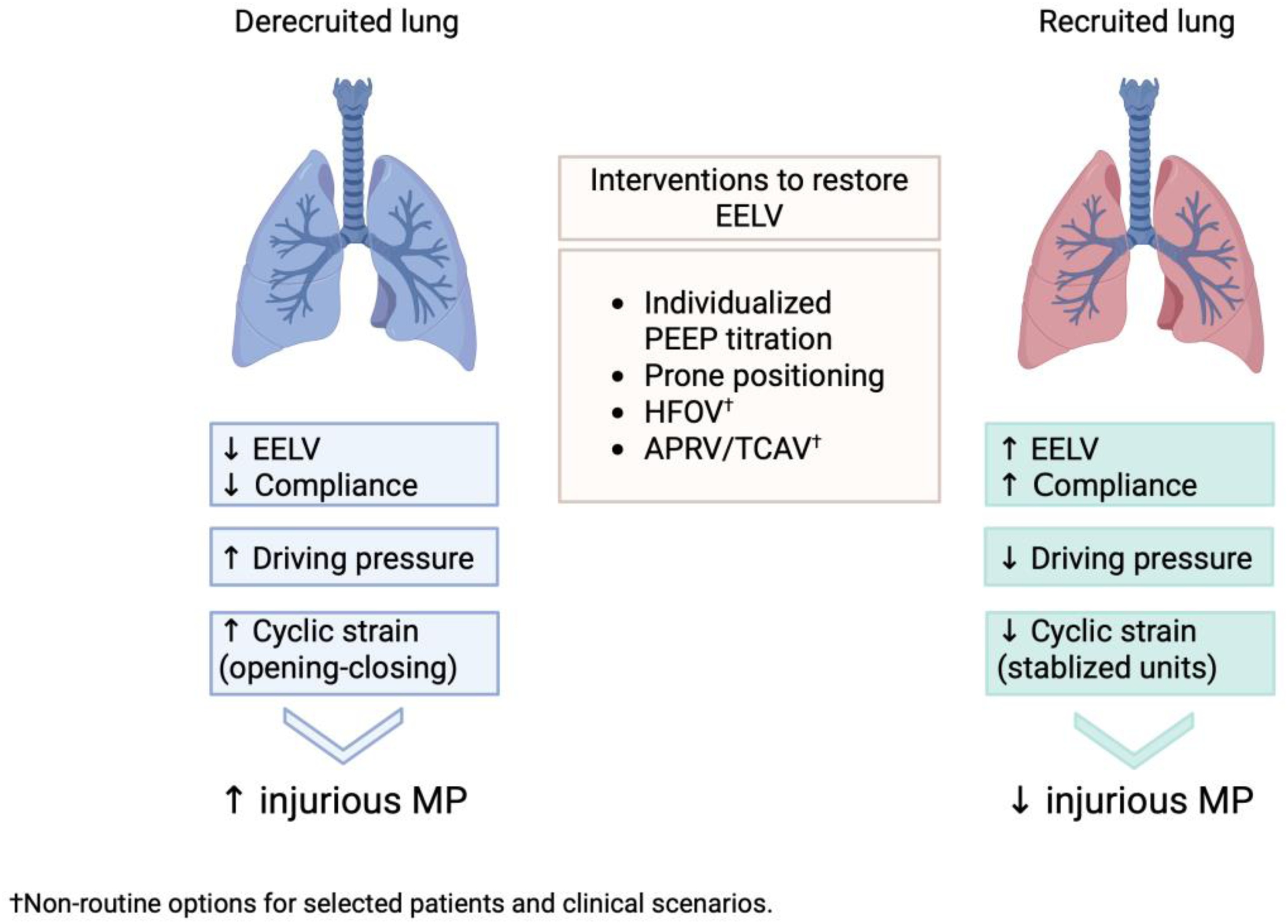
The figure illustrates how derecruitment with reduced end-expiratory lung volume (EELV) may decrease respiratory system compliance and increase driving pressure (ΔP), thereby amplifying cyclic strain through repetitive opening–closing of unstable lung units and promoting conditions associated with more injurious energy delivery during ventilation. Interventions aimed at restoring or preserving EELV—such as individualized PEEP titration and prone positioning—may increase EELV, improve compliance, reduce ΔP, and stabilize alveolar units, thereby potentially mitigating ventilation-related stress. Recruitment maneuvers are not included in the figure, in accordance with current ARDS guidelines, which support higher PEEP strategies without routine lung recruitment maneuvers and recommend against prolonged high-pressure recruitment maneuvers in patients with moderate-to-severe ARDS. HFOV and APRV/TCAV are shown as non-routine options for selected patients and clinical scenarios. APRV = airway pressure release ventilation; EELV = end-expiratory lung volume; HFOV = high-frequency oscillatory ventilation; MP = mechanical power; PEEP = positive end-expiratory pressure; TCAV = time-controlled adaptive ventilation

When mechanical power remains high despite conventional protective ventilator settings, reduced EELV may represent a central and potentially modifiable therapeutic target, not because its restoration necessarily lowers absolute mechanical power, but because it may improve the mechanical conditions under which ventilatory energy is delivered [30,31]. Loss of EELV contributes to reduced respiratory system compliance, higher driving pressures, and greater cyclic deformation of fragile lung units [[30], [31], [32]]. Clinically, this pattern may be suggested by reduced compliance, elevated driving pressure, marked oxygenation dependence on PEEP, and worsening mechanics following circuit disconnections or other derecruiting events [22,30,31]. Fig. 3 summarizes this physiological framework.

Reduced respiratory system compliance should not automatically be attributed to the lung alone, because increased chest wall elastance and intra-abdominal pressure may elevate plateau and driving pressure while also promoting derecruitment and reduced EELV [33]. When this contribution is suspected, respiratory mechanics should be interpreted cautiously and, where feasible, complemented by esophageal and intra-abdominal pressure assessment [33]. Strategies aimed at restoring EELV, particularly higher PEEP, also require attention to haemodynamic tolerance, as improved lung mechanics may be offset by adverse cardiovascular effects in vulnerable patients [34,35].

A physiologically plausible bedside response is individualized PEEP titration, ideally guided by assessment of recruitability rather than by oxygenation or global respiratory mechanics alone [26,36,37]. However, this approach should be considered a patient-specific physiological strategy rather than a universally outcome-proven intervention. In this context, PEEP should be viewed not as a generic escalation step, but as a means of stabilizing functional aerated lung volume and reducing repetitive derecruitment, provided that overdistension and haemodynamic intolerance are avoided [31,36,37]. This does not necessarily reduce absolute mechanical power, because higher PEEP may increase static energy load even when compliance improves and driving pressure falls [31]. Rather, the aim is to improve the mechanical conditions under which ventilatory energy is delivered by limiting cyclic collapse, strain concentration, and unstable lung mechanics [26,31]. The recruitment-to-inflation ratio may help identify patients more likely to benefit from higher PEEP [36,37].

Recruitment maneuvers may reopen collapsed lung units, but their value depends on whether the gained lung volume can be maintained without overdistension [[38], [39], [40]]. Accordingly, negative trials of recruitment-oriented strategies and high frequency oscillatory ventilation should be interpreted as highlighting the risk of non-individualized or overly aggressive approaches rather than negating the physiological relevance of EELV restoration [[41], [42], [43]]. Practical emphasis should therefore remain on selective, physiology-guided PEEP optimization rather than on routine use of recruitment maneuvers or other recruitment-oriented strategies [36,37,39,40].

Prone positioning may support EELV stabilization, although its dominant protective effect is improved stress redistribution and ventilation homogeneity [29,44]. Airway pressure release ventilation (APRV), particularly when configured using a time-controlled adaptive ventilation (TCAV) strategy, remains a mechanistically plausible but still incompletely validated strategy for gradual lung-volume stabilization [45,46]. Avoidance of derecruiting events, careful suctioning, appropriate sedation and, when indicated, neuromuscular blockade, as well as conservative fluid management, may further help preserve EELV and reduce the mechanical burden imposed on the lung [22,47,48].

**Fig. 4:**
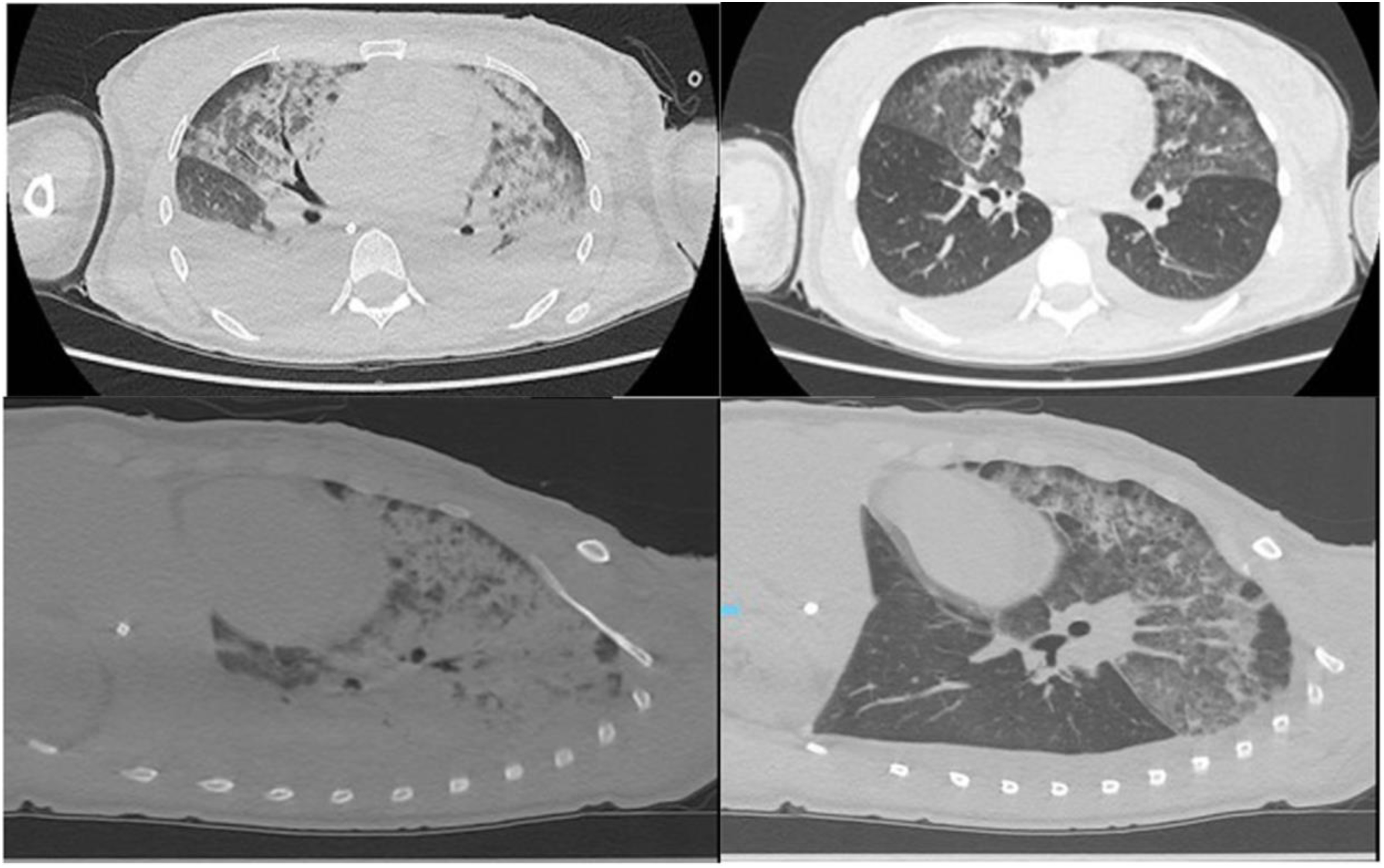
The image highlights that recruitment-oriented strategies are clinically meaningful only when they produce effective re-aeration and stabilization of previously collapsed lung regions, rather than simply exposing the lung to higher airway pressures. Adapted from Barbas et al., Frontiers in Physiology 2022 [64], under CC BY 4.0.

A representative computed tomography example of recruitment-guided lung re-aeration is shown in Fig. 4.

## Strategies targeting redistribution and homogenization

Regional lung heterogeneity is a key determinant of how harmful mechanical power becomes, because under the same global ventilator settings energy may be distributed unevenly within the lung [49,50]. Some regions may therefore be exposed to disproportionate stress, strain, and dissipative losses despite apparently acceptable global values [[49], [50], [51]].

**Fig. 5:**
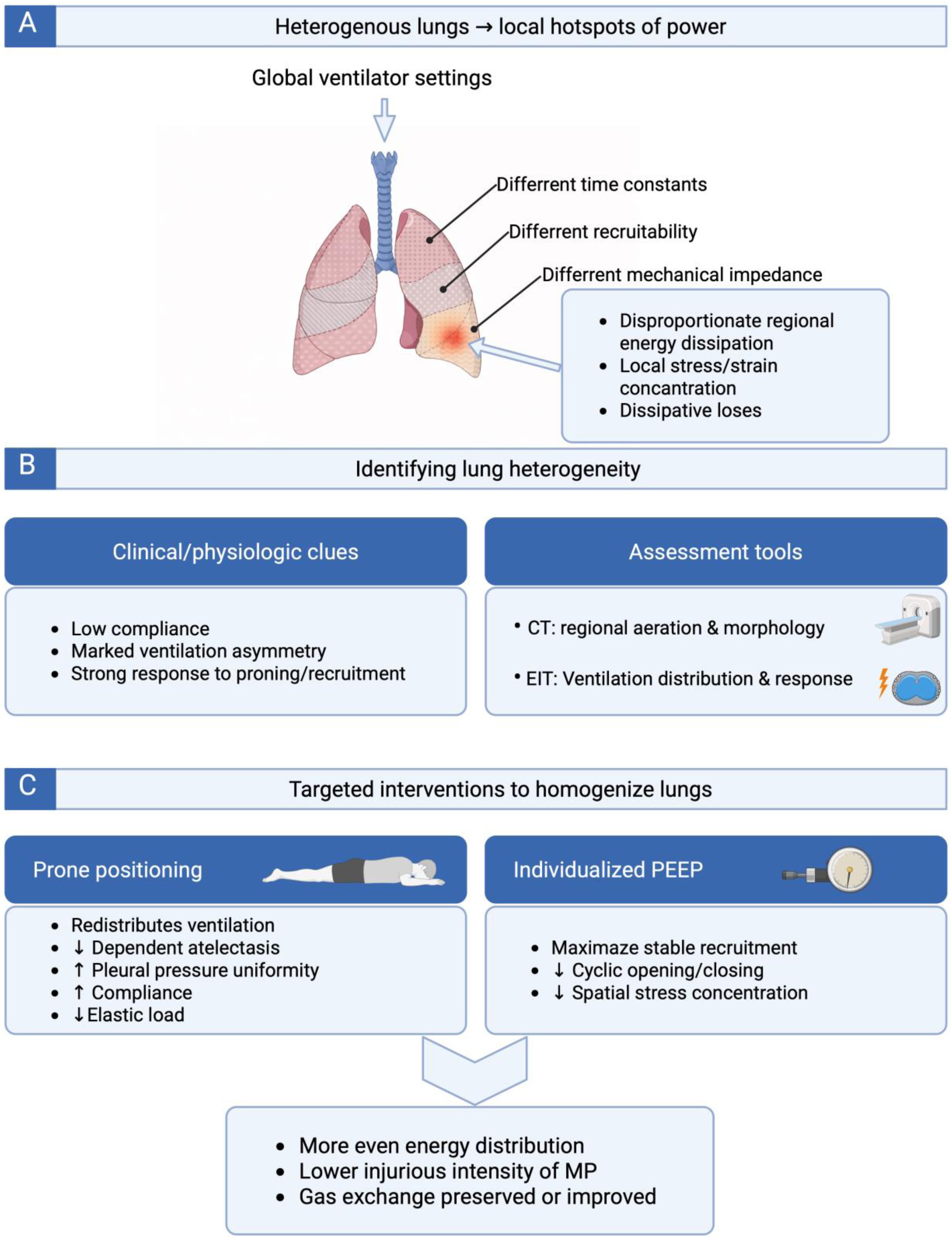
(A) Heterogeneous lungs are fragmented into compartments with different time constants, recruitability, and mechanical impedance. Under identical global ventilator settings, energy delivery and dissipation become spatially uneven, creating local hotspots of power characterized by disproportionate regional power delivery, local stress/strain concentration, and increased dissipative losses—despite nominally acceptable global ventilator metrics. (B) Lung heterogeneity should be suspected when clinical/physiologic clues suggest uneven ventilation (e.g., low compliance, marked ventilation asymmetry, or a strong response to prone positioning or recruitment). Regional assessment tools, including CT and bedside EIT, can reveal heterogeneity in aeration and ventilation distribution and quantify responses to changes in position or airway pressure that may remain occult to global parameters. (C) Targeted interventions aimed at improving homogeneity—particularly prone positioning and individualized PEEP—may redistribute ventilation, stabilize recruitment, reduce cyclic opening/closing and regional stress concentration, and thereby promote a more even regional energy distribution and preserve or improve gas exchange. CT = computed tomography; EIT = electrical impedance tomography; PEEP = positive end-expiratory pressure; MP = mechanical power.

Differences in time constants, recruitability, and impedance mean that the same ventilator settings do not affect all lung regions equally, favoring late opening, early overdistension, and repeated cyclic stress in selected units [49,50]. Imaging, especially computed tomography, and bedside tools such as electrical impedance tomography can help identify uneven aeration and ventilation distribution that are not visible from global ventilator parameters alone [21,25]. These patterns and their clinical implications are summarized in Fig. 5.

In this context, prone positioning has the strongest clinical support among currently available interventions in severe ARDS [27,28,44]. Its protective effect should not be interpreted primarily as direct reduction of mechanical power per se, but rather as redistribution of transpulmonary pressure, improved ventilation homogeneity, reduction of dependent atelectasis, and attenuation of regional stress raisers [[27], [28], [29],^44^]. By contrast, other approaches discussed in this review—particularly individualized PEEP titration, recruitment-oriented strategies, or APRV/TCAV—should be interpreted primarily as physiologically motivated, patient-specific, and hypothesis-guided adjuncts rather than interventions with equally established clinical evidence for reducing VILI.

Beyond its role in restoring global lung volume, individualized PEEP titration guided by lung recruitability assessment, ideally using imaging-derived or functional bedside tools such as EIT, represents another practical intervention to homogenize lung mechanics [26]. Setting PEEP to maximize stable recruitment while avoiding overdistension minimizes cyclic opening and closing and reduces spatial stress concentration [26]. Fixed-number PEEP tables do not achieve this reliably; targeted, patient-specific PEEP selection can reduce dissipated energy and improve its spatial distribution even when tidal volume, respiratory rate, and thus global mechanical power remain unchanged.

The preceding sections refer primarily to controlled ventilation, where the bedside framework focuses mainly on elastic constraints related to reduced functional lung volume and regional heterogeneity. During assisted ventilation, however, interpretation must additionally account for patient-generated inspiratory effort, dyssynchrony, and flow-related dynamic stress amplification.

## Modulating neural respiratory drive to reduce dynamic components of mechanical power

In assisted ventilation, mechanical power cannot be interpreted solely from ventilator settings and passive respiratory mechanics. Patient-related factors, particularly neural respiratory drive and inspiratory effort, may substantially augment inspiratory flow, pleural pressure swings, and transpulmonary pressure, thereby increasing the dynamic and potentially injurious component of the mechanical load applied to the lung [7,8,52]. This is especially relevant in lungs with reduced end-expiratory lung volume or marked heterogeneity, where vigorous effort may concentrate stress and strain within unstable regions [9,53]. Accordingly, apparently acceptable or only moderately elevated mechanical power values should be interpreted cautiously when there is evidence of excessive respiratory drive or strong inspiratory effort.

Where feasible, neural respiratory drive and inspiratory effort should be objectively assessed rather than inferred solely from ventilator waveforms. It is useful to distinguish drive, as neural output from the respiratory centers, from effort, as the pressure generated by the inspiratory muscles, because these may diverge under different loading conditions.

Airway occlusion pressure at 0.1 s (P0.1) is a practical bedside index of respiratory drive [54]. It reflects the early inspiratory pressure generated against an occluded airway and may help identify excessive central respiratory output. However, it is not a direct measure of inspiratory effort and may be influenced by ventilator-specific measurement algorithms and technical factors (e.g., whether P0.1 is measured during a true occlusion or estimated from the trigger phase, trigger sensitivity/timing, circuit-related factors, and the presence of leaks), as well as by intrinsic PEEP, operating lung volume, and neuromechanical coupling [[54], [55], [56]].

Tidal esophageal pressure swing (ΔPes) may serve as a surrogate of inspiratory effort and pleural pressure change [57]. It can help detect vigorous breathing, although its interpretation depends on appropriate signal acquisition and clinical context [57].

Within the present framework, P0.1 and ΔPes should not be viewed primarily as simple add-ons for calculating a corrected numerical mechanical power value at the bedside. Rather, they serve as complementary indicators that determine whether ventilator-derived mechanical power is likely to underestimate the true injurious mechanical burden imposed on the lung [7,53,58]. Elevated P0.1 suggests excessive neural respiratory drive, whereas large ΔPes indicates substantial patient-generated inspiratory effort and pleural pressure swing [54,57].

In practical terms, patient effort should be incorporated into mechanical power interpretation not by deriving a single corrected bedside value, but by reclassifying the significance of ventilator-derived mechanical power according to the presence and magnitude of respiratory drive and effort markers. Thus, a mechanical power value that might appear acceptable during passive or minimally assisted ventilation should be regarded as potentially injurious when accompanied by elevated P0.1, large ΔPes swings, marked tachypnea, dyssynchrony, or other signs of vigorous inspiratory effort. Conversely, the same ventilator-derived mechanical power is less concerning when these amplifying factors are absent. In this way, mechanical power during assisted ventilation is best interpreted as part of a two-domain bedside assessment: ventilator-delivered energy and patient-generated stress amplification.

Together, these measures support a more clinically meaningful interpretation of mechanical power during assisted ventilation: not by providing a precise corrected number, but by identifying when patient effort amplifies transpulmonary stress, inspiratory flow, pendelluft, and regional energy concentration beyond what is apparent from ventilator settings alone. Importantly, although pendelluft may plausibly increase local stress, strain, and energy dissipation, there is currently no well-established or clinically applicable way to quantify its contribution within the mechanical power framework [7,9]. It should therefore be interpreted primarily as a mechanism of regional stress amplification rather than as a directly measurable increment in bedside mechanical power.

High inspiratory effort may be driven by several factors, including severe hypoxemia, hypercapnia, metabolic acidosis, discomfort, pain, anxiety, and patient–ventilator dyssynchrony [52]. However, hypoxemia per se is not invariably associated with increased respiratory effort, and a robust association is mainly observed in severe hypoxemia [52,59].

Therapeutic strategies should therefore primarily target the underlying cause of increased respiratory drive, rather than indiscriminately suppressing patient effort [52]. Sedation alone may reduce respiratory effort numerically but may adversely affect lung mechanics when associated with derecruitment or prolonged controlled ventilation [9].

In selected phases of acute ARDS—particularly during the early exudative phase—short, targeted neuromuscular blockade may be appropriate to mitigate injurious pendelluft-related intracycle lung deformation, thereby reducing localized energy dissipation [9]. Restoration of EELV through prone positioning, appropriate PEEP titration, or APRV/TCAV may indirectly attenuate excessive neural drive by improving oxygenation and reducing the mechanical and neuromechanical load of breathing [9,53,60]. The goal is therefore to preserve beneficial spontaneous breathing when possible while preventing vigorous effort that amplifies dynamic lung stress [53,61].

## Extracorporeal approaches when mechanical power cannot be safely reduced

Despite optimized conventional lung-protective settings and adjunctive physiological strategies, mechanical power may remain at levels that cannot be safely sustained in some patients. In such cases, the limitation may reflect not inadequate ventilator settings, but the intrinsic inability of the injured, functionally small, and mechanically fragile lung to tolerate further energy delivery.

In this context, extracorporeal support should be viewed primarily as a means of maintaining gas exchange while allowing further reduction of ventilator intensity when this cannot otherwise be achieved safely. The physiological rationale for VV ECMO includes extracorporeal oxygenation and CO₂ removal, thereby permitting the native lungs to be ventilated with more conservative settings [62].

By providing extracorporeal CO₂ removal and, when needed, oxygenation support, ECMO can facilitate further reductions in tidal volume, driving pressure, and respiratory rate, thereby enabling reduction of ventilator-delivered mechanical power and external energy transfer to the lung [63]. However, ECMO should not be interpreted as reducing the lung’s intrinsic susceptibility to injury. Rather, its potential protective value is indirect: it may create conditions in which less injurious ventilatory support can be applied while lung recovery proceeds. Thus, the clinical rationale for extracorporeal support in this setting is not that ECMO itself makes the lung less vulnerable, but that it may permit safer ultra-protective ventilation when adequate gas exchange cannot otherwise be maintained without persistently high ventilatory intensity [62,63].

The practical bedside response to persistently elevated mechanical power is summarized in Box 1, which synthesizes the proposed physiological reasoning into a simplified clinical decision framework.

## Box 1. Bedside decision framework for persistently elevated mechanical power and increased lung stress during controlled and assisted ventilation

This framework is intended to support bedside physiological reasoning rather than serve as an evidence-graded clinical guideline. The strength of evidence differs substantially across interventions, and each step requires individualized clinical reassessment.

## Step 1. Define the ventilatory context

Determine whether ventilation is controlled or assisted, as the interpretation of mechanical power and lung stress differs fundamentally between these settings.

## Step 2A. Controlled ventilation: identify constraint-driven mechanical power

Confirm that conventional lung-protective targets have been achieved, including tidal volume ≤6 mL/kg predicted body weight, plateau pressure ≤28–30 cmH₂O, driving pressure ≤14–15 cmH_2_O when feasible, and no unnecessary elevation of respiratory rate or inspiratory flow.

Mechanical power should be considered constraint-driven when:

(1) conventional lung-protective limits have already been reached; and

(2) further reduction in ventilatory intensity, including tidal volume, respiratory rate, or PEEP when clinically appropriate, is reproducibly poorly tolerated, with worsening oxygenation, hypercapnia, respiratory acidosis, or derecruitment.

In practice, this transition is recognized by consistent deterioration during ventilatory de-escalation, often accompanied by persistently elevated driving pressure despite low tidal volume or clear PEEP dependence.

## Step 2B. Assisted ventilation: recognize when measured mechanical power may underestimate lung stress

Assess whether patient-generated inspiratory effort may amplify lung stress despite acceptable ventilator settings and measured mechanical power.

If excessive respiratory drive is present, mechanical power should not be interpreted in isolation, as transpulmonary stress and dynamic energy delivery may be substantially higher than estimated.

## Step 3A. Controlled ventilation: identify the dominant physiological constraint

Prioritize the mechanism that most directly explains poor tolerance of further ventilatory reduction.  

**Reduced or unstable end-expiratory lung volume (EELV)**

Suspect when deterioration follows PEEP reduction or derecruiting events, and when mechanics or oxygenation are strongly PEEP-dependent.

**Primary action**: prioritize PEEP optimization and strict avoidance of derecruitment, while monitoring for overdistension and hemodynamic compromise.  

**Regional heterogeneity and uneven stress distribution**

Suspect when low compliance persists despite apparently adequate lung volume, especially with evidence of asymmetry, strong response to prone positioning, or imaging/EIT signs of heterogeneous aeration.

**Primary action**: prioritize prone positioning to improve homogeneity and reduce regional stress; adjust PEEP if it enhances recruitment without overdistension.  

**Practical prioritization rule:**

If deterioration is triggered mainly by loss of lung volume, address EELV first.

If mechanics remain poor despite adequate volume and heterogeneity is evident, prioritize stress redistribution.

If both mechanisms coexist, treat the dominant one first and reassess sequentially.

## Step 3B. Assisted ventilation: identify excessive inspiratory effort

Prioritize this when spontaneous breathing generates potentially injurious lung-distending stress.

Indicators include elevated P0.1, large esophageal pressure swings, dyssynchrony, or persistent tachypnea.

**Primary action**: correct reversible drivers of respiratory effort, including hypoxemia, hypercapnia, acidosis, pain, anxiety, and patient–ventilator mismatch, and reduce injurious effort rather than indiscriminately suppressing spontaneous breathing.

## Step 4. Consider extracorporeal support when lung stress remains unavoidable

If adequate gas exchange cannot be maintained without persistently high ventilatory intensity in controlled ventilation or ongoing injurious inspiratory effort in assisted ventilation, despite targeted interventions, consider extracorporeal support to enable safer ultra-protective ventilation.

## Conclusions

Persistently elevated mechanical power despite optimized conventional lung-protective settings should be interpreted primarily as a marker of physiological constraint rather than simply a target for further reduction of ventilatory intensity. In this setting, the key clinical task is to identify the dominant modifiable mechanism that makes further reduction in ventilatory intensity poorly tolerated—most importantly loss of end-expiratory lung volume, regional heterogeneity, excessive inspiratory effort, or persistent gas-exchange demands.

At the bedside, this implies that mechanical power should not be interpreted only as a numerical target to be lowered. Rather, it should prompt consideration of why a given amount of energy is being delivered under potentially harmful mechanical conditions. When these constraints cannot be sufficiently corrected with conventional ventilatory strategies, extracorporeal support may be considered to facilitate ultra-protective ventilation.

## Authors' contributions

TU conceived the review concept and structure, performed the targeted literature search and evidence selection, and drafted the manuscript. MD contributed to the conceptual framework and interpretation of the physiological implications, and substantively revised the manuscript for important intellectual content. TU and MD reviewed and approved the final submitted version and agree to be accountable for their individual contributions and to ensure that questions related to the accuracy or integrity of any part of the work are appropriately investigated and resolved.

## Consent for publication

Not applicable.

## Ethics approval and consent to participate

Not applicable.

## Declaration of Generative AI and AI-assisted technologies in the writing process

Generative artificial intelligence (ChatGPT with the GPT-5.5 Thinking model, OpenAI, San Francisco, CA, USA) was used as a basic writing assistant to support language polishing and stylistic refinement of the manuscript. The AI tool was not used for the planning, conduct, or analysis of the study, nor to generate data, references, or figures, and it did not contribute to scientific interpretation or decision-making. All scientific content, interpretations, and conclusions remain the sole responsibility of the authors.

## Funding

This work was fully funded by the 10.13039/501100004442National Science Centre, Poland, under Grant No. 2023/50/A/ST7/00498.

## Availability of data and materials

Not applicable.

## Declaration of competing interest

The authors declare that they have no competing interests.
